# Optical triggered seizures using a caged 4-Aminopyridine

**DOI:** 10.3389/fnins.2015.00025

**Published:** 2015-02-04

**Authors:** Mingrui Zhao, Laura M. McGarry, Hongtao Ma, Samuel Harris, Jason Berwick, Rafael Yuste, Theodore H. Schwartz

**Affiliations:** ^1^Department of Neurological Surgery, Brain and Mind Center, New York Presbyterian Hospital, Weill Medical College of Cornell UniversityNew York, NY, USA; ^2^Department of Biological Sciences, Columbia UniversityNew York, NY, USA; ^3^Department of Psychology, University of SheffieldSheffield, UK

**Keywords:** caged compound, epilepsy model, neocortex, electrophysiology, optical imaging, photostimulation

## Abstract

Animal models of epilepsy are critical not only for understanding the fundamental mechanism of epilepsy but also for testing the efficacy of new antiepileptic drugs and novel therapeutic interventions. Photorelease of caged molecules is widely used in biological research to control pharmacologic events with high spatio-temporal resolution. We developed a technique for *in vivo* optical triggering of neocortical seizures using a novel caged compound based on ruthenium photochemistry (RuBi-4AP). Epileptiform events in mouse cortex were induced with blue light in both whole brain and focal illumination. Multi-electrode array recording and optical techniques were used to characterize the propagation of these epileptic events, including interictal spikes, polyspikes, and ictal discharges. These results demonstrate a novel optically-triggered seizure model, with high spatio-temporal control, that could have widespread application in the investigation of ictal onset, propagation and to develop novel light-based therapeutic interventions.

## Introduction

Epilepsy is a disease that affects about 1% of the population in the United States involving recurrent seizures, or ictal events (Thurman et al., 2011). The lifelong effects of chronic epilepsy can be devastating due to progressive cognitive decline from short duration electrical discharges that occur throughout the epileptic network between seizures called interictal events. Moreover, uncontrolled seizures cause increases in patient morbidity and mortality. The cost to society is enormous (Begley et al., 2000; Wilner et al., 2014). The mechanism of neocortical epilepsy is poorly understood (Navarro et al., 2002). Animal models of epilepsy are very important not only for understanding the fundamental mechanisms of epilepsy but also for testing the efficacy of new antiepileptic drugs or other therapeutic interventions (Löscher, 2011). New neuroimaging and neuro-modulatory techniques, such as optogenetics have emerged as a popular tool to probe and control neuronal activity with light. Recently, focal seizure-like afterdischarges have been induced in rat hippocampus using optogenetics (Osawa et al., 2013). Caged compounds, also called phototriggers, are widely used in biological research. Photorelease of caged molecules can be used to administer neurotransmitters and other chemicals with high spatial-temporal resolution. However, the ability of such caged compounds to trigger seizures and interictal events has not been previously investigated.

4-Aminopyridine (4-AP) is a relatively selective blocker of voltage-activated K^+^ channels and a potent convulsant when applied to the neocortex. Focal 4-AP application to the neocortex *in vivo* generates tonic-clonic ictal focal electrographic seizures, which respond to all standard anticonvulsants (Bruckner and Heinemann, 2000). In the *in vitro* slice model, 4-AP is released into the bath solution, thus influencing the whole slice in a generalized way. With bath application, often in association with removal of magnesium from the bath (Yang et al., 2010), seizure-like events can initiate in multiple locations and multiple layers, but in an uncontrolled fashion. *In vivo*, on the other hand, microinjection of 4-AP can induce focal seizures, but the volume of tissue in which the 4-AP is released is difficult to control and seizures initiate from only one location. The ability to non-invasively induce focal seizures in restricted areas of cortex with precise control of the timing of these events does not currently exist either *in vivo* or *in vitro*.

Caged compounds consist of a chemical entity composed of two parts: the caged compound of interest and a cage moiety that inhibits its action (Mayer and Heckel, 2006). Upon irradiation, the compound of interest is freed and can interact with the surrounding media. A spatial resolution of <0.5 μm and a temporal resolution of ns-to-ms are ideally achievable with focal fast photolysis. Recently, a new group of caged compound based on metal coordination chemistry was developed to study brain function (Rial Verde et al., 2008; Salierno et al., 2010; Araya et al., 2013). Ruthenium bipyridyl complexes can undergo ligand substitution when irradiated with visible light, without the production of a radical species.

In this manuscript we examine and describe a new technique for triggering seizures and interictal spikes used caged 4-AP (Nikolenko et al., 2005). This new technique, in contrast to the technique previously employed in our laboratory, namely focal injection of 4-AP (Zhao et al., 2011), allows the possibility of precise spatiotemporal control of the initiation and intensity of the ictal or interictal event allowing the investigator to address a variety of new questions with increased experimental control.

## Materials and methods

### Animal preparation

All experimental procedures were approved by the Weill Cornell Medical College Animal Care and Use Committee following NIH guidelines. Adult male CD1 mice (6–8 weeks) were anesthetized and maintained stable at normal values as previously described (Zhao et al., 2009).

### Acute epilepsy model

Adult CD1 mice were used for these experiments after approval was obtained by the IACUC of Weill Cornell Medical College. Mice were induced with isoflurane (2–4%) in 70% N_2_:30% O_2_ by facemask. After induction, the animal was maintained under isoflurane anesthesia using a small mask at ~1.5%. Animals were mounted in a stereotaxic frame. The heart rate, arterial blood oxygen saturation, and end-tidal CO_2_ were monitored and maintained at normal values throughout the experiment. Temperature was monitored rectally and maintained at 37°C with a homeothermic blanket system (Harvard Apparatus, Holliston MA). A ~ 4 × 5 mm cranial window was opened over one hemisphere between Lambda and Bregma to expose the neocortex. The dura was carefully removed. Ruthenium-bipyridine-triphenylphosphine caged 4-aminopyridine (RuBi-4-AP) (200 μl, 10 mM) was topically applied or microinjected into neocortex at least 30 min before uncaging. For the focal illumination, 2 μl RuBi-4-AP solution was injected in neocortex at the depth of 300 μm using an UltraMicroPump (UMP-3, WPI, Sarasota, FL) via a glass electrode controlled at a speed of 100 nl/min. For control experiments, 4-AP ictal discharges were induced by injecting 4-AP (Sigma, 15 mM, 0.5 μl) at the same location using a Nanoject II injector as previously described (Schwartz and Bonhoeffer, 2001; Zhao et al., 2009).

### Photostimulation

**Figure 1:**
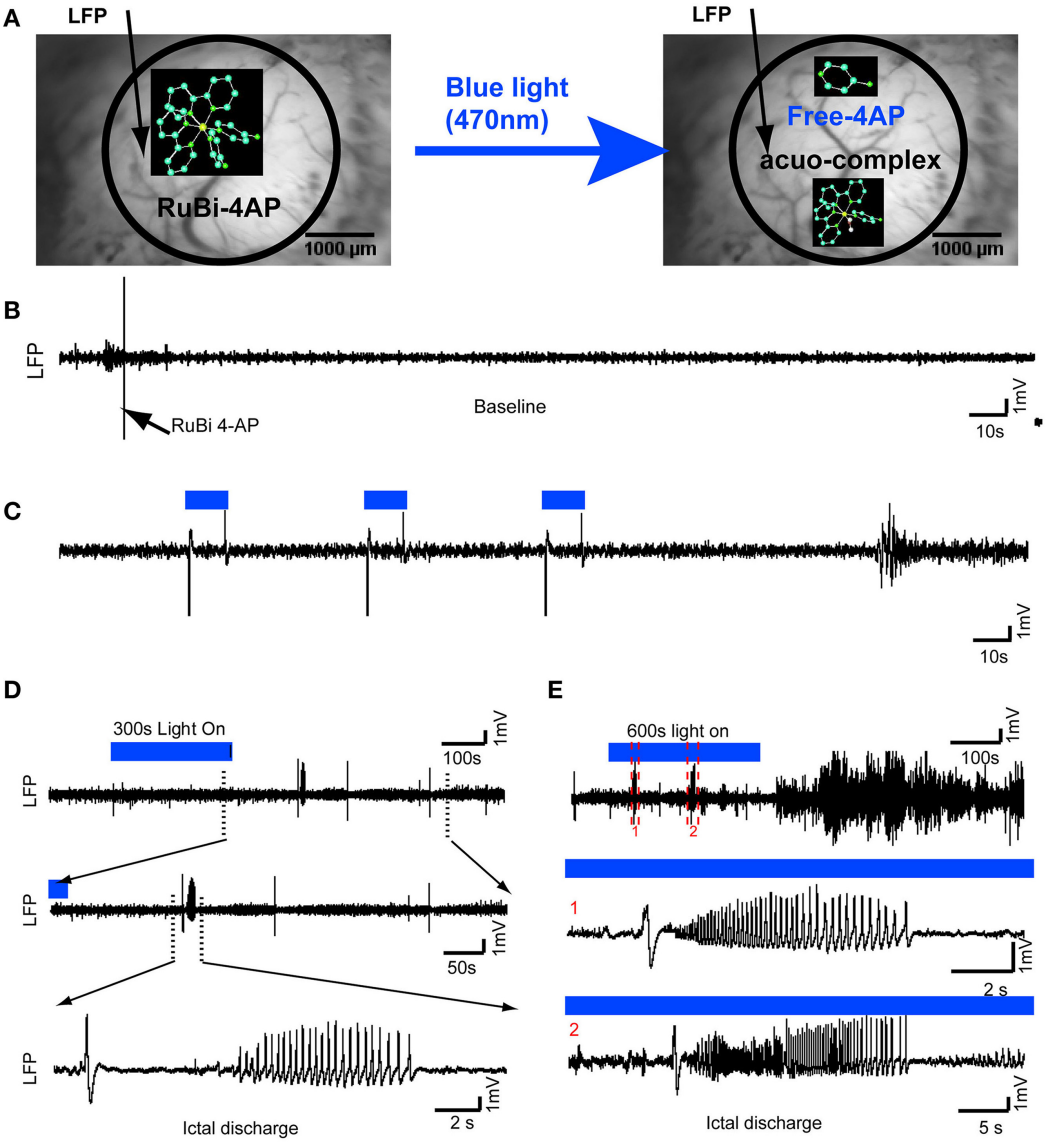
**Optical triggering of epilepsy using topical application of RuBi-4-AP *in vivo***. **(A)** Schematic diagram of experimental design. RuBi-4-AP (0.1 ml, 15 mM) is topically applied to induce seizures. RuBi-4-AP is stable in ACSF solution (Left) and the 4-AP and ruthenium complex are photoreleased with visible 470 nm blue light (Right). The black arrow on the image of the cortical surface indicates the LFP electrode. **(B)** 5 min baseline LFP recording shows normal brain activity after RuBi-4-AP application before uncaging. **(C)** Interictal discharges and polyspikes are induced by 10 s blue light illumination (470 nm, 1.2 A) over the neocortex. The duration of illumination is shown with a blue line (Top). **(D)** Ictal discharge is induced by 300 s blue light pulses. The expanded view (Bottom) shows a typical ictal discharge starting with an initial spike (ictal onset). **(E)** Ictal-like activity is also induced by longer, 600 s, illumination. Ictal discharges are recorded during illumination and even after the blue light is turned off (Top). The middle (1) and bottom panels (2) show expanded views of two ictal discharges in the upper panel.

Rub-4-AP can be uncaged with either 1 photon excitation (λ = 480 nm) or 2-photon excitation (800 nm) (Nikolenko et al., 2005; Fino et al., 2009). Uncaging was performed with 470 nm LED light (Figure 1A, Thorlabs, Newton, NJ) either broadly to the entire cortex or focally, delivered through an optical fiber (OD: 200 μm, Thorlabs, Newton, NJ), using a high power LED driver (LEDD1B, Thorlabs, Newton, NJ), controlled by a stimulator (Master-8; AMPI, Jerusalem, Israel). Optical power delivered at the fiber tip was calibrated with a PM100USB power meter (Thorlabs, Newton, NJ).

### *In vivo* electrophysiology

A single channel local field potential (LFP) was recorded by placing a glass electrode (impedance, 2–4 MΩ) filled with 0.9% saline into the cortex at an acute ~45° angle. The LFP was amplified and filtered between 0.1 and 1000 Hz using an AC/DC Differential Amplifier (Model 3000, A-M Systems, Carlsborg, WA). The signal was then digitized by a CED Power 1401 (Cambridge Electronic Design, Cambridge UK), and recorded onto a PC using Spike as previously described (Zhao et al., 2009). In some experiments, multi-channel depth electrodes were employed for multi-layer recordings (16 channels with 100 μm spacing, site area 177 μm^2^, 1.5–2.7 MΩs impedance, and 33 μm tip width; Neuronexus Technologies, Ann Arbor, MI), coupled to a preamplifier and data acquisition device (RZ5D workstation, TDT, Alachua, FL) using RPvdsEx software (TDT, Alachua, FL) (Harris et al., 2013).

### Intrinsic optical spectroscopic imaging

The exposed cortical surface was illuminated with one of two different wavelengths using two high power LEDs either at 530 nm (M530L2; 1600 mA;Thorlabs, Newark, NJ) or 780 nm (M780L2, 1000 mA; Thorlabs, Newark, NJ). The light was provided through a liquid light-guide placed ~20 mm away from the cortex. Reflected light was collected through a tandem lens assembly using two 50 mm camera lenses. Signals recorded at 530 nm, an isosbestic wavelength for hemoglobin, provide a measure of total hemoglobin (Hbt), which is proportional to cerebral blood volume (CBV) if the hematocrit remains stable (Grinvald et al., 1986; Frostig et al., 1990). At 780 nm, the majority of the optical signal is derived from the scattering of light caused by a variety of factors including cell swelling and intra- and extra-cellular fluid shifts, which provide an indirect spatial representation of neuronal activity (Salzberg et al., 1985). Images were acquired at 10 Hz (Imager 3001, Optical Imaging Inc.) as previously described (Zhao et al., 2011). A TTL signal from each image is routed to an Arduino microcontroller (Arduino Diecimila, Sparkfun Electronics, Boulder, CO), which is programmed to serially strobe the two LED drivers, alternating between the two wavelengths.

### Data analysis

Offline analysis was performed using custom analysis software written in Matlab (MathWorks, Natick, MA). Reflectance changes at 530 and 780 nm were expressed as a negative percentage change from the baseline (−ΔR/R). Each frame following the onset of each seizure, measured with the LFP, and for 20 s before onset, was divided by an average of the 20 frames (2 s) prior to the onset. An average of the percent fractional change in the optical signal, positive peak, and negative peak at each time point were used for further analysis. The time interval between the ictal onset and the time point where the optical signal crossed 2SD beyond the baseline recording was used to measure the latency of optical signal. For epileptic events, total LFP (ΣLFP) power was calculated as an integral of the area above 2 SD baseline activity under the response curve of each event. Baseline activity was measured over a 2 s epoch prior to the initiating spike for epileptic events or 10 s before/after light illumination. A longer window was used prior to illumination since the changes would be more subtle and this would permit an increase in the signal to noise ratio.

For all data, statistical significance was determined with Student's *T*-test. All data were expressed as means ± standard error of mean (SEM).

## Results

### Optical triggering of epilepsy using topical application of RuBi- 4-AP

4-AP is a potent convulsant (Rogawski and Barker, 1983; Stansfeld et al., 1986; Mattia et al., 1993; Barkai et al., 1995; Benardo, 1997). In previous experiments, we have shown that 4-AP is a powerful convulsant when injected into the neocortex of *in vivo* anesthetized rat and mouse (De la Cruz et al., 2011; Zhao et al., 2011). In order to test whether the caged compound RuBi-4-AP would induce abnormal brain activity prior to photorelease and to ensure that the illumination alone would not elicit abnormal cortical activity, we performed the following control experiments. Once the surgery was complete, we recorded baseline LFP signals for 15 min to ensure the health of the neocortex and the absence of any abnormal activity that might result from the trauma of surgery. Animals (5%) with abnormal spikes or bursts induced by surgical damage before RuBi-4-AP application were excluded from the further experiments. Blue light was applied to the cortex, 20 mm from its surface, for a period of 600 s. No abnormal spikes, bursts or DC shifts were identified. In fact, the illumination appeared to have no effect on the LFP after comparing the power spectrum of the LFP before and after illumination (averaged ΣLFP_power_ in 10 s: 38.57 ± 21.27 mV^2^ vs. 36.25 ± 13.83 mV^2^, *n* = 4 mice, *p* > 0.05). This control experiment demonstrates that the induced epileptic activity was not caused by light damage or any other photostimulation effect. RuBi-4-AP (15 mM, 200 μl) was then topically applied on the neocortex for 30 min. In the absence of blue light illumination (no uncaging), there were no abnormal spikes, bursts or DC shifts detected and no significant change in the power spectrum of the LFP for all recorded mice (Figure 1B). This result indicated that the caged compound was stable without blue light illumination and when not uncaged, did not trigger any type of epileptiform events.

### Wide-field illumination

We used a high power 470 nm LED light (1.2 A) to uncage RuBi-4-AP over an exposed cortical region in anesthetized mice. The duration of illumination could be used to trigger different types of events, either interictal spikes or ictal events. Using short 10-s pulses, interictal spikes were reliably induced (Figure 1C). The average time between short-duration illumination and interictal events was 0.90 ± 0.21 s (*n* = 4 mice, 268 interictal spikes after 120 photostimulations). Following longer duration illumination (300–600 s), seizure-like ictal discharges were triggered (Figures 1D,E). These seizures typically began with a large spike-and-wave followed by a low amplitude fast activity, or recruiting rhythm, which evolved into rapid spike-and-wave activity that gradually increased in periodicity and decreased in amplitude until the seizure terminated. Consistent with our previous 4-AP experiments using focal injection of the drug (Ma et al., 2013), some seizures began with low-voltage fast activity without the initial spike. Even after illumination was discontinued, ictal-like events were continuously recorded for up to 3100 s, indicating that the uncaged drug was persistently active and did not become re-caged. The morphology of these events is similar to those elicited with focal 4-AP injection in rat neocortex and also human neocortical seizures (Zhao et al., 2007, 2009). In total, 268 interictal spikes (*n* = 4 mice) and 34 ictal discharges were recorded (*n* = 4 mice). The average duration of seizures was 39.41 ± 11.57 s. The average delay between the onset of long-duration illumination and a first ictal event was 303.10 ± 151.55 s.

### Focal illumination

**Figure 2:**
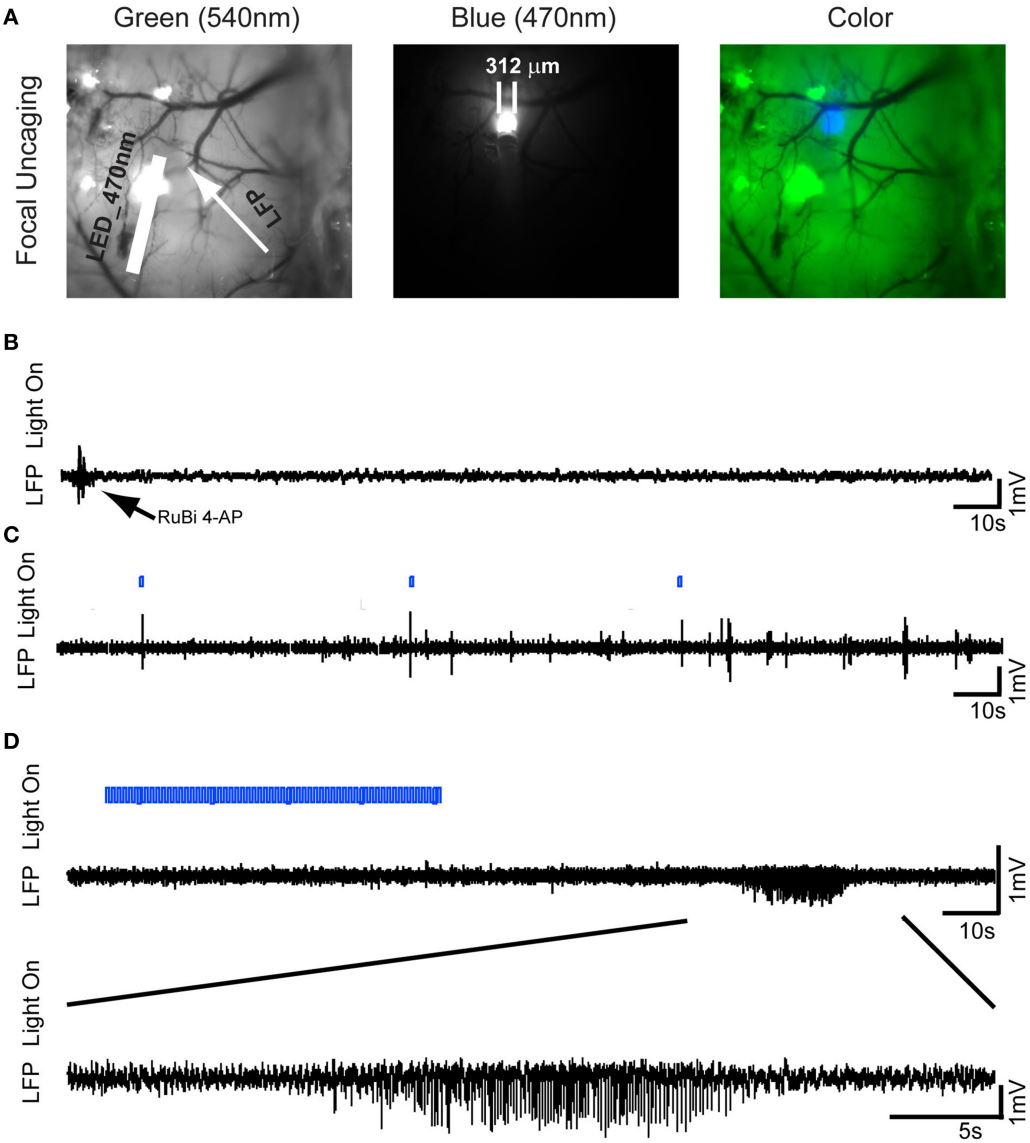
**Optical triggering of focal epilepsy using focal illumination and RuBi-4-AP *in vivo*. (A)**RuBi-4-AP (2 μl, 25 mM) is injected into the neocortex to induce seizures. RuBi-4-AP is photoreleased with an optical fiber (470 nm LED). The white arrow on the image of cortical surface indicates the LFP electrode and the white bar shows the optical fiber. Left: green light; Middle: blue light; and Right: merged image. **(B)** Baseline recording shows normal brain activity after RuBi-4-AP application before uncaging. **(C)** LFP recording (black) with light on marker. Interictal discharges and polyspikes are induced by 1 s focal blue light. The blue bar shows the LED light-on period. **(D)** One ictal discharge is induced by 60 s blue light illumination (Top). The further expended view (Bottom) shows an ictal-like discharge induced by focal blue light illumination.

After demonstrating the ability to trigger seizures with a large, diffuse uncaging, we wished to determine if focal uncaging was possible, which would permit spatial control of the site of onset of the seizures. Two μl of 25 mM RuBi-4-AP solution were injected into the neocortex at the depth of 300 μm using a MicroPump via a glass electrode (100 nl/min). With an optical fiber (200 μm OD) touched on the brain surface, we illuminated a focal area ~ 312 μm OD to induce the local photorelease of 4-AP (Figure 2). As with the topical RuBi-4-AP application, there was no abnormal brain activity without blue light illumination after focal RuBi-4-AP injection (Figure 2B). However, repeated 1 s short duration blue light stimulations elicited interictal spikes and polyspikes. The average time from the start of the stimulation after such a short pulse to the occurrence of an interictal spike was 2.34 ± 1.69 s. Longer light illumination (600 s) induced seizure-like ictal discharges (Figures 2C,D). Additional photostimulations were given if there were no ictal discharges in 5 min. We recorded 38 ictal discharges using focal illumination (*n* = 5 mice.) The average duration of seizures was 13.81 ± 5.33 s. The average time it took to record a seizure after a longer duration illumination onset was 638.32 ± 138.82 s (See each mouse's ictal data in Supplementary Material Table S1).

**Figure 3:**
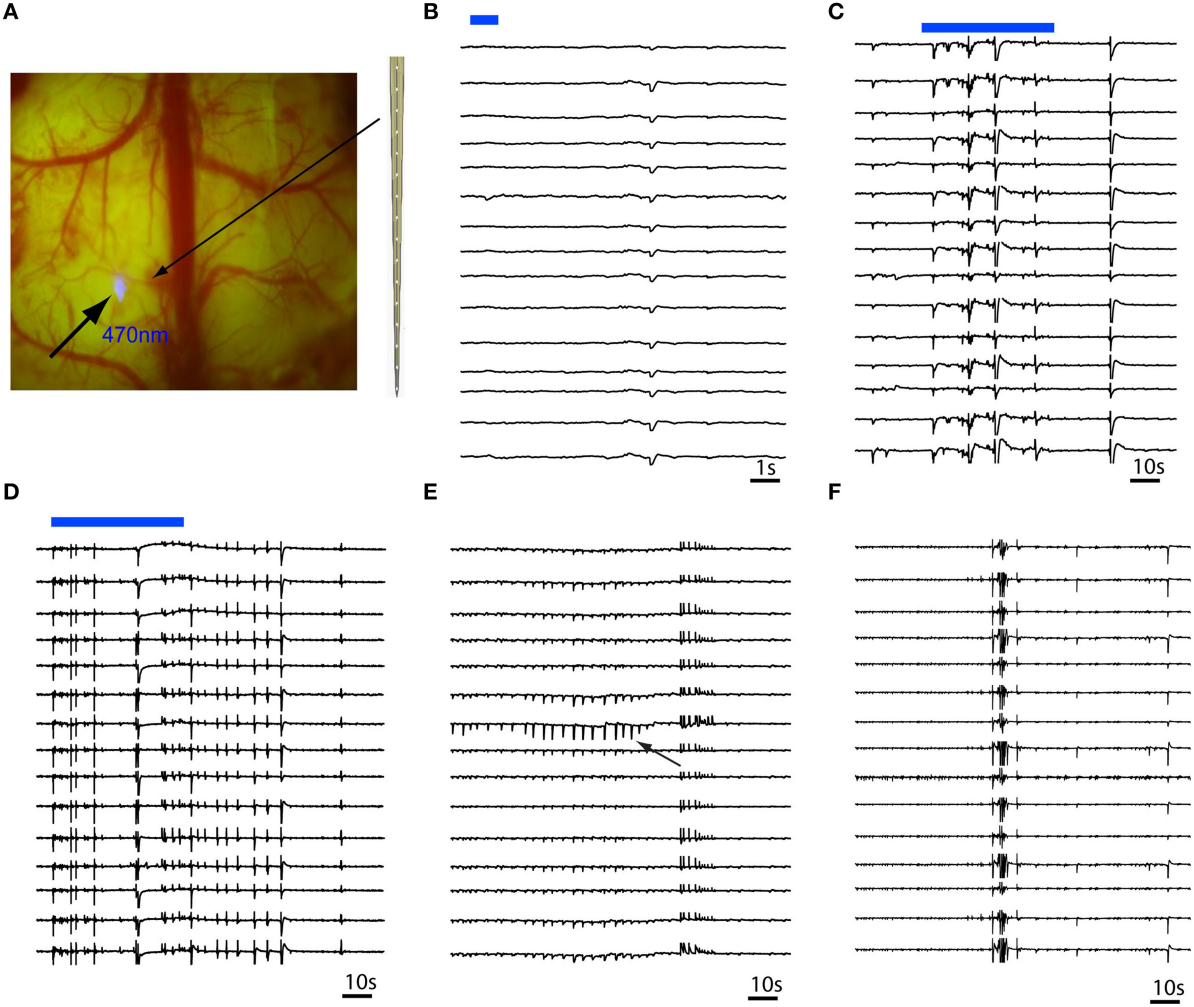
**Multi-contact laminar depth array recording of *in vivo* RuBi-4-AP epileptic events**. The depth electrode is placed 0.2 mm away from the site of focal illumination. **(A)** Focal photostimulation 0.2 mm away from the laminar depth electrode placed tangentially into the cortex. **(B)** A 1 s pulsed photostimulation (blue line) results in a short duration event that occurred simultaneously in all layers. **(C,D)** Longer duration illumination (60 s) results in immediate onset of polyspikes and short duration events involving all layers simultaneously. **(E)** Occasionally, layer specific events (black arrow) occur that quickly spread to all other layers. **(F)** However, the majority of events involve all layers simultaneously. The blue bars in **(B,C)** show the timing of blue photostimulation.

In order to investigate whether focal illumination of the cortical surface would trigger events that start at the surface or deeper in lower cortical layers, we recorded RuBi-4-AP events using a multi-contact laminar depth electrode (Figure 3A). In the presence of RuBi-4-AP, a short 1 s duration pulse of photostimulation resulted in small events that were not full-formed interictal spikes but appeared to be elicited by a small population of neurons exhibiting a post-synaptic excitatory event (Figure 3B). Longer duration illumination (60 s) resulted in the immediate onset of polyspike events occurring in all layers simultaneously that did not evolve into full blown seizures (Figures 3B–D). Layer-specific events sometimes occurred after the illumination was terminated and these events often exhibited field effects in all cortical layers (Figure 3E). However, most events involved all layers simultaneously (Figure 3F). Similar events were recorded in all animals (*n* = 4 mice). Of all the events (*n* = 103 events, 4 mice) recorded, 10.68% were layer-specific and 89.32% involved all layers simultaneously. Of the layer-specific events, all first started from layers IV to V and then propagated to the other neocortical layers.

### Optical mapping of RuBi-4-AP ictal discharge

**Figure 4:**
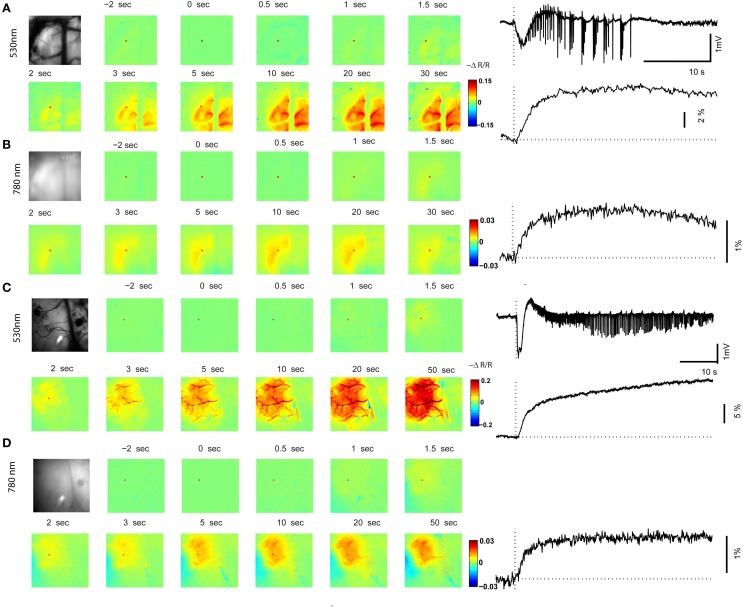
**Optical mapping of *in vivo* ictal discharge**. **(A,B)** 530 nm map and 780 nm map of a focal ictal discharge induced by RuBi-4AP and photostimulation. The left panel shows an image of the cortical surface and images at selected time points with respect to seizure onset (0 s). The right panel shows time course of the LFP (local field potential), CBV (cerebral blood volume), and 780 nm signals recorded during one seizure. Red dots in the images are the location of the ROIs in the seizure focus. **(C,D)** 530 nm map and 780 nm map of a focal ictal discharge induced by 4-AP. The left panel shows an image of the cortical surface and images at selected time points with respect to the seizure onset. The right panel shows the time course of LFP, CBV, and 780 nm signals during one seizure. Red dots in the images are the locations of the ROIs in the seizure focus.

In order to determine if neurovascular coupling and propagation of the RuBi-4-AP events were similar to those caused by direct 4-AP injection, we performed optical spectroscopic mapping of the triggered events and compared them with maps derived from direct 4-AP injection. Optical recording of CBV at 530 nm during RuBi-4-AP ictal events demonstrated a monophasic increase in CBV (Figure 4A), with a mean latency of 0.82 ± 0.53 s. The mean duration of CBV increase was 9.58 ± 1.74 s. The maximum amplitude of focal CBV increase was 5.56 ± 0.95% (*n* = 5 mice, 30 ictal discharges). At 780 nm, the maps of light scattering demonstrated a monophasic increase as well but of smaller amplitude and spatial spread (Figure 4B). The mean duration of 780 nm signal increase was 8.38 ± 2.57 s. The maximum amplitude of focal 780 nm signal increase was 2.27 ± 0.16% (*n* = 5 mice, 30 ictal discharges). Both CBV and light scattering imaging showed a clear optical response to RuBi-4-AP ictal events.

**Figure 5:**
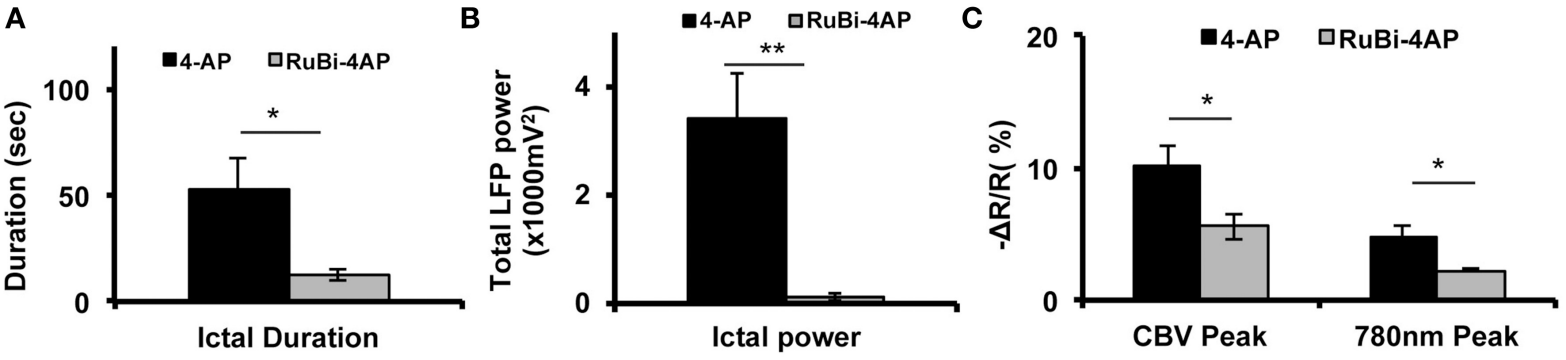
**CBV and light scattering response to ictal discharges elicited by RuBi-4-AP or 4-AP**. **(A)** Average seizure duration induced by RuBi-4-AP is significantly shorter compared to seizure duration induced by 4-AP (*p* < 0.05). **(B)** Average total LFP power of seizures induced by RuBi-4-AP was significantly decreased compared with the total LFP power of seizures induced by 4-AP (*p* < 0.01) **(C)** Average maximum response of CBV and 780 nm signals in the seizure focus was significantly decreased in the RuBi-4-AP compared with the 4-AP seizures (CBV: *P* = 0.04; 780 nm: *p* = 0.023). ^*^p < 0.05; ^**^p < 0.01.

In order to compare RuBi-4-AP-induced seizures with seizures induced by injected standard 4-AP (Figure 5), we injected 0.5 μl 4-AP (15 mM) into the neocortex as described previously (Zhao et al., 2011). The power of the resulting LFP seizure was 122.79 ± 63.93 mV^2^ (*n* = 5 mice, 30 ictal discharges) with RuBi-4-AP and 3414.75 ± 836.83 mV^2^ (*n* = 5 mice, 57 ictal discharges) with 4-AP. Average seizure power induced by RuBi-4-AP was significantly lower than seizure power induced by injected 4-AP (*p* < 0.01). The morphology of the CBV and light scattering responses to RuBi-4-AP were similar to injected 4-AP but smaller in size and amplitude, as would be expected based on the size of the electrographic events (Figures 4C,D). The maximum amplitudes of focal CBV increase and 780 nm maps resulting from injected 4-AP were 10.12 ± 1.63 and 4.78 ± 0.88%, respectively (*n* = 5 mice, 57 ictal discharges), which was significantly larger than the RuBi-4-AP amplitudes (*p* < 0.05; Figures 5B,C). The average duration of injected 4-AP ictal discharges was 53.02 ± 14.56 s, which was significant longer than the average duration of RuBi-4-AP seizures (*P* < 0.05; Figure 5A). Thus, the hemodynamic and metabolic patterns of the RuBi-4-AP events indicate preserved neurovascular coupling, undamaged by any phototoxicity.

## Discussion

In this study, we provide, for the first time, evidence that photorelease of caged 4-AP can trigger epileptic events including interictal spikes, bursts of polyspikes, and ictal discharges of varying morphologies and durations. This model can be induced *in vivo* with both whole brain and focal illumination to provide high spatio-temporal control of the site of ictal onset. We describe two separate techniques for using of RuBi-4-AP and provide the foundation for other uses. With global application and wide-field illumination both interictal spikes and full-blown ictal events can be triggered. The former occur within seconds whereas the latter take a few minutes to develop. The location of the onset of these events is multifocal but the technique is non-invasive since the drug is applied topically. The variability in the resulting morphological of the electrographic events that can be triggered reflects the myriad of electrographic patterns manifested in clinical epilepsy (Prince, 2014). With focal injection, and focal illumination, the site of onset can be controlled more precisely. The amplitude and duration of these events vary based on the duration of illumination and can even occur in single layers, which provides not only the ability to investigate the development of epileptic events in a graded fashion but the occurrence of microseizures, which have been shown to occur in humans and whose synchronization may be critical to the development of human ictal events (Schevon et al., 2008; Stead et al., 2010). Recent data indicated that seizure initiation and spread is not highly hyper-synchronous (Truccolo et al., 2011). Using RuBi-4-AP, the ability to alter the concentration of the drug as well as the size and amplitude of the illumination can lead to an almost infinite number of possibilities, which can be tuned to create precisely the desired type of epileptiform event. As such, the full range of combinatorial events is beyond the scope of this paper, as are the possibilities available with 2-photon illumination to trigger seizures in even smaller microdomains and specific layers as discussed below. As we have shown, this model is also useful to investigate neurovascular coupling as the amplitude and duration of events are shorter than would be produced with direct focal injection. Likewise, the resulting amplitude and duration of the associated vascular events are smaller, indicating proportional relationship in epileptiform neurovascular coupling, consistent with the existing literature (Bruehl et al., 1998; Ma et al., 2009; Geneslaw et al., 2011).

### Optically-triggered seizures

New neuroimaging and neuromodulatory techniques, such as optogenetics, which combine optical and genetic methods, and optochemistry, which involve the photorelease of active drugs, have emerged as popular tools to probe and control neuronal function with light (Zemelman et al., 2002; Lima and Miesenböck, 2005; Petreanu et al., 2007; Szobota et al., 2007; Zhang et al., 2007; Zayat et al., 2011; Chow and Boyden, 2013). Recently, optical suppression of epilepsy has been studied using both optogenetic techniques as well as caged compounds (Tonnesen et al., 2009; Yang et al., 2010). Optogenetics involves a gene manipulation, while caged methods involve a pharmacological manipulation of a chemical so that light can trigger the release of the chemical. Unlike optogenetic methods, the photochemical approach does not require gene modification, which means the manipulation is reversible.

Acute rodent models of neocortical epilepsy include focal injection and topical application of pharmacologic agents. Focal GABAergic blockade is effective at creating interictal events *in vivo* but full blown seizures rarely occur (Schwartz and Bonhoeffer, 2001; Suh et al., 2005). 4-AP is a potent convulsant when applied to the neocortex. The microinjection of 4-AP can induce focal seizures, but the ability to trigger multiple sites of onset is limited and the seizures begin at a specific time after injection that cannot be controlled once the drug is injected. The possibility of optical triggering of interictal and ictal events offers several advantages in seizure control and modeling. Two methods currently exist for possible optical triggering of seizures, optogenetics and uncaging. While optogenetics holds certain advantages, such as the ability to target specific cell-types and both open and close channels thereby providing a reversible trigger (Fenno et al., 2011), the majority of research in the field has focused on seizure termination rather than seizure induction. Although unclear at this time, it may be that the specificity of optogenetics is too fine to reliably elicit seizures, which often requires a perturbation in a large network of cells. Recently, however, seizure-like afterdischarges were elicited optogenetically in *in vivo* hippocampus (Osawa et al., 2013). Uncaging, on the other hand, introduces a pharmacologic agent non-specifically which can influence the network at multiple levels and in multiple cell types. While less specific, this manipulation may be more efficacious at achieving the goal of epileptogenesis. It is also possible to induce focal seizures in deep brain regions, such as the hippocampus using optical fibers. Furthermore, unlike 4-methoxy-7-nitroindolinyl- (MNI) or 1-(2-nitrophenyl)ethoxycarbonyl (NPEC) group caged compounds, which require UV light (<360 nm), RuBi- caged chemicals can be photo-triggered by visible blue light (470 nm), which has a higher tissue penetration and is less damaging to the living tissue. RuBi-4-AP also has a higher quantum efficiency of uncaging (Nikolenko et al., 2005). The lack of cell specificity is also an advantage at seizure termination, which has been successfully performed using RuBi-GABA (Yang et al., 2010, 2012).

### Microseizures and lamina-specific seizures

The ability to use RuBi-4-AP to elicit microseizures in multiple limited cortical domains can be aided with the development of light-delivery technology. One such technology, micro-LED arrays, permits multisite photostimulations (Grossman et al., 2010). Likewise, two-photon uncaging has been successfully used to activate individual neurons and dendritic spines (Fino et al., 2009). The application of multisite photostimulation in the RuBi-4-AP model will provide the ability to model multiple microseizures. Using fine resolution microelectrode arrays in human epileptic tissue has identified a new class of electrographic seizures localized to sub-millimeter-scale tissue, at the level of the cortical column, labeled “microseizures” (Schevon et al., 2008; Stead et al., 2010). The existence of microseizures suggests that epileptiform activity can occur within extremely small neuronal networks. Microseizures play an important role in the initiation of epileptic seizures as small localized events appear to coalesce into full-blown seizures (Dudek, 2009). Most importantly, multiple small microseizures can occur in multiple regions of cortex either simultaneously or sequentially. Currently there is no model of microseizures in existence to investigate this novel phenomenon and RuBi-4-AP provides such a technique.

Using a depth laminar array electrode, we have also demonstrated that RuBi-4-AP can trigger laminar-specific epileptiform events. Data from *in vitro* slice preparations and *in vivo* human experiments have suggested that seizures may initiate in layer 5 and propagate in layers 2–3 (Gutnick et al., 1982; Silva et al., 1991; Sutor et al., 1994; Chesi and Stone, 1998; Telfian and Connors, 1998; Ulbert et al., 2004; Jin et al., 2006). Other studies, however, have raised the hypothesis that epileptiform events can be formed in several (probably all) neocortical layers or may arise from hyperconnected neurons in layer 2/3 neurons (Tsau et al., 1999; Beaumont et al., 2012). Preliminary analysis of our data indicates that even with topical release of RuBi-4-AP, all layer-specific events occurred in infragranular layers.

How can release of 4-AP in supragranular layers trigger seizures in layers 4 and 5? To estimate the release of caged chemicals, it is important to understand the transmission of visible light in brain tissue. Measurements and theoretical calculations of blue light penetration have been previously done in the brain tissue (Adamantidis et al., 2007; Aravanis et al., 2007; Huber et al., 2008; Yizhar et al., 2011). In our multi-contact laminar electrode array experiment, the tip of optical fiber was put on the top of exposed cortex. Due to the light absorption and scattering in brain tissue, the light-induced release of RuBi-4AP would be relatively well-localized at the surface of neocortex. Most neocortical neurons (70–80%) are excitatory pyramidal neurons and the remaining 20–30% are interneurons, mostly inhibitory interneurons (Markram et al., 2004). Layer 1 consists primarily of the distal tufts of pyramidal cell apical dendrites, a few of GABAergic neurons, and many axon terminations. More specifically, the excitatory cells in layer 4 and 5 have their dendrites in the superficial layers. Layer 1 is a major target of -feedback- connections between associated cortical areas (Douglas and Martin, 2004). We hypothesize that topical release of 4-AP acts on the dendrites of the pyramidal cells in layers 4 and 5. Excitatory neurons in the neocortex use recurrent excitatory feedback as an effective network conductance to amplify external network inputs (Douglas et al., 1995). These large pyramidal neurons in layer 5 typically respond with bursting discharge patterns (Stoop et al., 2002). The epileptic discharges may then be triggered by increasing recurrent excitatory connections.

In conclusion, we provide a novel optically-triggered rodent epilepsy model using a caged chemical. Although the precise control of this RuBi-4-AP model is still under development, we have already shown that epileptiform events including interictal spikes, polyspikes, and ictal discharges were induced using a visual-range wavelength light illumination. Both multisite LFP recording and optical mapping showed clear seizure-like activity. Further development of this model *in vivo* and *in vitro* will be useful to address specific questions regarding epileptogenesis and neurovascular coupling.

## Author contributions

Mingrui Zhao, Rafael Yuste and Theodore H. Schwartz generated the research idea, study design and concept. Mingrui Zhao, Rafael Yuste, and Theodore H. Schwartz wrote the manuscript. Mingrui Zhao, Laura M. McGarry, Hongtao Ma, and Samuel Harris performed experiments. Mingrui Zhao, Jason Berwick, Rafael Yuste, and Theodore H. Schwartz analyzed and interpreted data.

### Conflict of interest statement

The authors declare that the research was conducted in the absence of any commercial or financial relationships that could be construed as a potential conflict of interest.
