# Supplementary material for: Optical triggered seizures using a caged 4-Aminopyridine
Source: Front Neurosci. 2015 Feb 4;9:25. doi: 10.3389/fnins.2015.00025 (PMC4316705; doi:10.3389/fnins.2015.00025)
Supplement: Supplementary file 1 [file Table1.DOCX]

**Table S1 Characterization of ictal events from wide field and vs focal stimulation**

| **Mouse #** | **Photostimulation** | **Ictal Events** | | **Average Ictal Duration (sec)** | | **Average ΣLFP_power_ (mV^2^)** | **Latency to 1st events (sec)** |
| --- | --- | --- | --- | --- | --- | --- | --- |
| 1 | wide field | 4 | 13.16±3.63 | | 40.16±14.84 | | 389.13 |
| 2 | wide field | 7 | 27.48±8.96 | | 30.54±8.09 | | 52.05 |
| 3 | wide field | 10 | 63.34±10.58 | | 103.10±19.44 | | 695.11 |
| 4 | wide field | 13 | 53.64±7.69 | | 22.75±4.28 | | 76.08 |
| 5 | focal | 8 | 9.26±3.49 | | 12.09±3.43 | | 500.11 |
| 6 | focal | 3 | 11.05±2.36 | | 21.19±6.92 | | 212.61 |
| 7 | focal | 10 | 34.82±6.50 | | 70.75±20.39 | | 614.5 |
| 8 | focal | 4 | 8.61±2.99 | | 3.54±1.85 | | 855.27 |
| 9 | focal | 13 | 5.32±0.28 | | 7.55±2.18 | | 1009.09 |

ΣLFP_power_: the total local filed potential power of ictal discharge.
